# Representation and social vulnerability to HIV in transgender people

**DOI:** 10.1590/0034-7167-2023-0353

**Published:** 2025-08-08

**Authors:** Karla Romana Ferreira de Souza, Elizandra Cassia da Silva Oliveira, Carla Andreia Alves de Andrade, Sérgio Corrêa Marques, Fátima Maria Leite Cruz, Aurélio Molina da Costa, Fátima Maria Abrão da Silva

**Affiliations:** I Universidade de Pernambuco. Recife, Pernambuco, Brazil; II Universidade Estadual do Rio de Janeiro. Rio de Janeiro, Rio de Janeiro, Brazil; III Universidade Federal de Pernambuco. Recife, Pernambuco, Brazil; IV Universidade de Leeds. Leeds, United Kingdom

**Keywords:** Nursing, Transgender Persons, Social Vulnerability, HIV, Social Theory, Enfermería, Personas Transgénero, Vulnerabilidad Social, VIH, Teoría Social

## Abstract

**Objectives:**

to analyze the social representations of HIV vulnerability in transgender women.

**Methods:**

this qualitative study was based on the Theory of Social Representations, using the structural approach. The research was conducted in a hospital specializing in gender-affirming surgeries, involving 100 self-declared transgender women. The Free Word Association technique was applied using the inducing term “HIV exposure in transgender women” to obtain representational content. Data analysis was performed using the four-quadrant technique, facilitated by the *Interface de R pour les Analyses Multidimensionnelles de Textes et de Questionnaires* software, version 0.7 alpha 2.

**Results:**

the possible central nucleus identified included the terms “prevention, prostitution as a profession, prejudice, and risky behavior”. Social and individual issues were highlighted as important elements in the vulnerability process.

**Conclusions:**

the social representations of HIV vulnerability in transgender women are grounded in structural factors that lead to inequality, exclusion, prejudice, and discrimination.

## RESUMEN

**Objetivos:**

analizar las representaciones sociales de la vulnerabilidad al VIH en mujeres transexuales.

**Métodos:**

estudio cualitativo, basado en la Teoría de las Representaciones Sociales, utilizando el enfoque estructural. La investigación se llevó a cabo en un hospital de referencia en cirugías de reasignación de género, con 100 mujeres autodeclaradas transexuales. Se utilizó la técnica de Evocaciones Libres de Palabras al término inductivo “exposición al VIH en mujeres transexuales” para la obtención de los contenidos de la representación. El análisis de los datos se realizó mediante la técnica del cuadro de cuatro casas, instrumentalizada por el *software Interface de R pour les Analyses Multidimensionnelles de Textes et de Questionnaires*, versión 0.7 alpha 2.

**Resultados:**

se identificaron, en el posible núcleo central, los términos “prevención, prostitución como profesión, prejuicio y comportamiento de riesgo”. Se destacaron cuestiones sociales e individuales como elementos importantes en el proceso de vulnerabilidad.

**Conclusiones:**

las representaciones sociales de la vulnerabilidad al VIH en mujeres transexuales se basan en factores estructurales que generan desigualdad, exclusión, prejuicio y discriminación.

## INTRODUCTION

The situations of vulnerability to the human immunodeficiency virus (HIV) are among the greatest challenges for controlling the epidemic, as they are considered responsible for its spread and impact various populations differently. Data from the Joint United Nations Program on HIV/AIDS (UNAIDS) in 2019 indicate that, globally, transgender populations have 12 times the risk of HIV infection compared to the general population^(1)^.

Studies on vulnerability seek to understand people’s perceived risk of HIV exposure and progression to Acquired Immunodeficiency Syndrome (AIDS), taking into consideration that this is not solely the result of individual factors but also of collective and contextual dimensions that heighten susceptibility to infection and illness. Additionally, access to resources varies across federal, state, and municipal levels, impacting the protection offered within these governmental spheres. Vulnerability analysis aims to integrate three interdependent dimensions: individual, social, and programmatic or institutional, enabling a deeper understanding of the aspects of people’s lives, communities, and nations that make them more or less susceptible to HIV infection, illness, or death from AIDS^(2,3)^.

It is important to emphasize that transgender women are often subjected to marginalization, exacerbated by overlapping prejudices related to their gender identity. This dynamic, characterized as transphobia, profoundly impacts their lives and health, placing them in a state of continuous social precarity within the health-disease process. Transphobia is not merely an isolated form of discrimination but rather a structural system that reinforces multiple forms of marginalization and inequality, significantly increasing vulnerability to HIV^(4)^.

Research on HIV vulnerability aims to analyze the epidemic’s spread and its impact on various populations. Its findings facilitate the identification and recognition of differences and specificities within this process, which are essential for planning and implementing policies and programs tailored to support the most vulnerable groups exposed to HIV^(5)^.

Currently, there is a lack of studies addressing HIV dissemination from the perspective of disease vulnerability and the constructed meanings used to confront this reality. Efforts focus on understanding the representational and consensual universe of transgender women in relation to HIV. Since HIV/AIDS is a social phenomenon that has undergone transformations over time, giving voice to those receiving healthcare is crucial for understanding their needs based on everyday experiences, attitudes, behaviors, and emotions. This understanding contributes to more effective and satisfactory health promotion and prevention efforts^(6,7)^.

Understanding the HIV/AIDS epidemic, which emerged in 1990, as an individual issue depends on the socioeconomic and cultural context in which the individual is situated. Discussions previously framed from the perspective of individual behavior classified certain groups as “at-risk populations”, a categorization that continues to generate social vulnerability, stigma, and prejudice. Today, these discussions have shifted toward issues of social vulnerability, enabling debates on human rights and health^(8)^.

The Theory of Social Representations (TSR), based on the principles of Moscovici, encompasses three different theoretical-methodological approaches for studying the phenomenon of Social Representations (SR): the processual (or culturalist) approach, developed by Denise Jodelet; the structural approach, proposed by Jean-Claude Abric; and the societal approach, advocated by Willem Doise^(9)^.

Reflecting on the situations of HIV vulnerability among transgender women may have important implications, especially concerning prevention within this population. The structural approach of the TSR, which corresponds to the central core theory developed by Jean-Claude Abric, facilitates linking the relationships that transgender women establish within their everyday practices and the meanings they attribute to the conditions of HIV vulnerability.

## OBJECTIVES

To analyze the SR of HIV vulnerability among transgender women.

## METHODS

### Ethical Aspects

This study was approved by the Research Ethics Committee and conducted in accordance with the guidelines of Resolution No. 466/2012 of the National Health Council. All participants signed an Informed Consent Form (ICF).

### Type of Study

This is a qualitative study conducted in accordance with the recommendations of the Consolidated Criteria for Reporting Qualitative Research (COREQ).

### Theoretical-Methodological Framework

This study is based on the TSR by Serge Moscovici, with an emphasis on the Central Core Theory (CCT). This theory comprises SR constructed through two main processes: anchoring and objectification. Anchoring involves the incorporation or assimilation of new elements of an object into a system of categories already familiar to the subject, which are available in memory. Objectification, on the other hand, seeks to transform what is abstract into something concrete, relating it to the physical world and linking unfamiliar concepts with reality^(9)^.

The theoretical-methodological approach used to study the phenomenon of SR was the structural approach proposed by Jean-Claude Abric. This approach examines the content of SR and how they are organized, considering issues of stability and change, as well as their relationship with the social practices of the group in question. There is a central core around which the SR is structured, determined by the nature of the object under study, the group’s relationship with it, and the system of social values and norms that comprise its ideological context. The central core is the most stable element of the representation and, therefore, resistant to change. There are also peripheral elements that make up the SR, which are organized around the central core. These elements are more accessible, dynamic, and concrete, integrating everyday experiences and being more individualized and context-specific^(10)^.

### Study Setting

The research was conducted at the Care and Support Center for Transgender People, located within a university hospital that serves as a reference center for gender-affirming surgeries in the North-Northeast region of Brazil. Invitations to participate in the study were extended in the hospital’s waiting area while transgender women awaited their appointments. Once the invitation was accepted, participants were taken to a private room designated by the institution for individual interviews.

### Data Source

The study included 100 self-declared transgender women. Participants were selected through convenience sampling, based on the following inclusion criteria: identifying as transgender and identifying as female. Engagement with participants was facilitated by the multidisciplinary team. Individuals with hearing impairments were excluded, as the researcher did not have proficiency in Brazilian Sign Language.

## RESULTS

The sociodemographic and clinical characterization of the participants revealed that the predominant age group was 18 to 29 years, accounting for 54 (54%) of the participants. Regarding educational attainment, 65 (65%) reported having completed high school. In terms of income, 44 (44%) stated that they had no income, and with respect to marital status, 70 (70%) reported being single.

The output from the free word associations reflects the structure of SR of HIV vulnerability among transgender women. The following parameters were defined for data processing by the software, which required prior establishment: the minimum word frequency was set at 11, excluding terms evoked with less frequency; the calculation of the average frequency of evocation, which was 28.8. Based on these frequencies, the program calculated the Mean Orders of Evocation (MOE), which was 2.88, adjusted to 2.9.

When evoking the social object “HIV exposure among transgender women” to understand it, other objects became interwoven with the central object of the research. This indicates that participants did not focus solely on the topic of HIV exposure among transgender women as prompted but instead related to the discussions through the lens of their life histories, bringing in other themes embedded within the process of HIV exposure among transgender women. This demonstrates that no representational object exists in isolation but is interconnected with other objects, as described in Chart 1.

**Chart 1:** Four-Quadrant Table on the inducing term “HIV Exposure among Transgender Women”, based on the program report

Minimum frequency: 11						
**Intermediate frequency: 28.8**						
**Average order: 11**						
**Average order < 11**				**Average order > = 11**		
**1^st^ Quadrant - Central Core**				**2^nd^ Quadrant - First Periphery**		
**F > = 28.8**	Prevention	60	2.8	Vulnerabilidade social	53	3.3
	Prostitution as a profession	46	2.7	Sofrimento intemo	52	3.1
	Prejudice	42	2.7			
	Risk behavior	34	2.9			
**3^rd^ Quadrant - Contrast Zone**				**4^th^ Quadrant - Second Periphery**		
**F < = 28.8**	Trying to be positive	27	2.7	Drugs	16	3.2
	Unprotected sex	23	2.8	Addiction	16	3.4
	Lack of job opportunities	13	2.8	Trust	15	3.1
	Weakness of public policies	12	2.7	Misinformation	12	3.8
	No family support	11	2.7			

*Source: Research data processed using IRaMuTeQ® software.*

The results from the report on the frequency of evocations (F), in response to the inducing expression “HIV exposure among transgender women”, are presented in the upper left quadrant, which displays the probable central elements-these are the words most frequently mentioned by transgender women and considered the most important: prevention, prostitution as a profession, prejudice, and risky behavior. The most frequently cited term, “prevention”, was mentioned 60 times (with a frequency of 2.8), followed by “prostitution as a profession”, mentioned 46 times (frequency of 2.7), “prejudice”, mentioned 42 times (frequency of 2.7), and “risky behavior”, mentioned 34 times (frequency of 2.9). The presence of these terms in the probable central nucleus of the SR regarding HIV exposure among transgender women reflects the importance placed on HIV prevention by participants. However, social vulnerability situations are recognized, considering that many of these women are pushed into prostitution as a profession due to prejudice. The presence of the term “risky behavior” demonstrates an acknowledgment of HIV exposure situations from the perspective of individual vulnerability.

Given these findings, it can be observed that all terms from the possible central nucleus are interconnected and suggest vulnerability situations to which transgender women are subjected in their daily lives. It is important to note that the absence of a definitive assertion regarding the central nucleus was due to the inapplicability of the centrality test to confirm the central nucleus, caused by time constraints for the research’s conclusion.

The peripheral system (first and second peripheries) comprises the following elements: in the first periphery, located in the upper right quadrant, “social vulnerability” was evoked 53 times (with a frequency of 3.3), and “internal suffering” was mentioned 52 times (with a frequency of 3.1). When evaluating the first periphery, the term “social vulnerability” stands out due to its high frequency (60), reinforcing the content present in the central nucleus, expressed through the terms “social vulnerability” and “internal suffering”, which refer to the affective-attitudinal dimension related to social vulnerability. The terms expressed in the second periphery present some negative attributes, such as “drugs”, “aidético” (a derogatory term for someone with AIDS), “trust”, and “disinformation”, referring to a more conceptual dimension as well as affective and attitudinal aspects, stemming from the historical and cultural construction surrounding HIV/AIDS and transgender women, relating to their individual vulnerabilities.

In the second periphery, located in the lower right quadrant, the most prominent elements are: drugs, aidético, trust, disinformation, sharp objects, needle contamination, lack of affection, mistrust, and kiss. The elements of greatest relevance are “drugs” and “aidético”, due to their higher frequencies and lower MOE in this quadrant. The term “drugs” is strongly linked to AIDS at the onset of the epidemic in Brazil, while the word “aidético”, commonly used early in the epidemic, gave rise to stigma and prejudice. These terms reflect the historical and cultural construction of the disease. Therefore, it can be stated that these elements represent the various meanings attributed by transgender women to HIV vulnerability.

The elements present in the contrast zone, located in the lower left quadrant, are terms with low frequency and low MOE, meaning they were mentioned less often but were quickly evoked by participants, and are thus considered important by the few who mentioned them. These include: “trying to be positive”, “unprotected sex”, “lack of job opportunities”, “fragility in public policies”, and “no family support”.

The term “trying to be positive” was the most prominent within the contrast zone, due to its higher frequency among the elements in this quadrant, highlighting the importance of coping mechanisms in facing vulnerability situations. The remaining terms reinforce and justify the elements evoked in the probable central nucleus.

## DISCUSSION

In the possible central nucleus of the SR regarding HIV exposure among transgender women, representational contents reveal one positive attribute related to the attitude of HIV/AIDS prevention and three negative attributes. The positive attribute pertains to the prevention of the disease, associated and conditioned by condom use during sexual practices. Conversely, the negative aspects are linked to sociocultural, economic, and psychosocial issues, expressed through the terms “prostitution as a profession”, “prejudice”, and “risky behavior”.

The central nucleus is composed of normative and functional elements. Thus, “prevention” is considered the functional dimension as it is associated with behaviors related to social practices. On the other hand, the elements “prostitution as a profession”, “prejudice”, and “risky behavior” are normative, as they are tied to the group’s historical and ideological context and the social value systems to which they belong. These elements prioritize judgments, stereotypes, and opinions accepted by the individual or social group within their context^(11)^.

Among the preventive elements, condom use during sexual relations was highlighted in a study conducted in Brazil with sex workers as the most known and utilized form of protection against HIV/AIDS^(12)^. However, combined prevention has become a new approach established by the Brazilian Ministry of Health for HIV prevention, including pre-exposure prophylaxis (PrEP) and post-exposure prophylaxis (PEP), as well as the distribution of male and/or female condoms, lubricating gels, regular HIV testing on demand, and counseling services^(13)^.

Contrast elements, such as “trying to be positive”, “unprotected sex”, “lack of job opportunities”, “fragility in public policies”, and “no family support”, were identified in the research results. Although less frequent, these terms are significant to some participants and relate to the central themes of prevention, prostitution as a profession, prejudice, and risky behavior. Transgender women acknowledge the need to overcome the challenges of surviving in a social context marked by transphobia, which deeply affects their right to citizenship. Promoting initiatives that restore self-esteem, rebuild support networks, and affirm safe life projects is crucial for them to maintain a positive outlook. This context of social vulnerability, exacerbated by transphobia, prejudice, lack of family support, and limited job opportunities, often leads to prostitution as a means of income, resulting in risky negotiations around condom use, leading to unprotected sex and generating risky behaviors. The fragility of public policies in promoting effective prevention measures specific to transgender women is evident^(14)^.

Therefore, discussing public policies to increase the availability of prevention strategies is both important and necessary, particularly in professional training. A study conducted in Brazil revealed a knowledge deficit among medical and nursing students regarding the use of PrEP and PEP, as also noted in studies conducted in Nepal, Ghana, and Ethiopia. Synergy is required to improve the quality of health services, particularly in professional training^(15)^.

The use of hormones in the process of body transformation can lead to erectile dysfunction, increasing the likelihood that a transgender woman assumes the receptive role in sexual relations. Evidence suggests that negotiating condom use can be challenging, as it often falls upon the man to decide whether or not to use protection. Unprotected anal sex poses a high risk of HIV transmission. Transgender women face significant challenges in advocating for condom use during sexual relations with men^(16)^.

Regarding the sociocultural, economic, and psychosocial issues faced by this population, many are rooted in a historical narrative that aims to codify heteronormative bodies. This often results in violence, invisibility, and vulnerability for individuals who diverge from established social norms. Such individuals are often viewed as anomalies, deviations, or illnesses due to their behavior, which raises numerous questions, discomfort, and resistance within society^(17)^.

Studies indicate that stigma and discrimination play a critical role, influencing behaviors, practices, and attitudes toward HIV. These factors can limit access to socioeconomic, educational, and employment resources, as well as access to prevention services, making them two of the main contributors to high HIV prevalence rates^(18)^. For instance, a study conducted in Argentina revealed that 40.7% of transgender women avoid using health services due to their gender identity, often stemming from experiences of discrimination by healthcare professionals or patients, and even police aggression^(19)^.

In the peripheral system of the analyzed SR, social vulnerability and internal suffering highlight the relationships among intersubjectivities, social contexts, dialogues, and conflicts that permeate the daily lives of transgender women concerning central elements. According to Abric^(10)^, these are the most accessible components of a representation and are the most susceptible to change. They update and contextualize normative and consensual determinations, facilitating an interface between concrete reality and the central system.

The transgender population faces numerous situations of social vulnerability, often characterized by exclusion from family and educational environments, typically marked by rejection and violence, leading to various internal struggles. According to the National Network of Trans People and the National Association of Transsexuals and Travestis of Brazil (ANTRA in Portuguese)^(20)^, 82% of transgender women drop out of high school between the ages of 14 and 18, and 90% end up in prostitution.

Transgender people are frequently excluded from various social groups, including their own parents, families, or society, due to their deviation from the cis-heteronormative system. This disruption of established gender norms leads to prejudice and discrimination, contributing to a range of negative effects on mental health. Data suggest that the suicide rate among cisgender individuals is approximately 4.6%, while the risk among transgender individuals rises to 41%. It is important to emphasize that youth from gender minorities represent a particularly vulnerable group when it comes to suicide^(21)^.

A study conducted in Brazil found that, among 154 transgender participants, 48.3% reported suicidal ideation, and 23.8% had attempted suicide, with higher levels of ideation and attempts observed among individuals with gender dysphoria compared to the general population. Similarly, studies conducted in Switzerland revealed that the prevalence of suicidal ideation was nearly seven and a half times higher among transgender respondents^(22)^.

The content and internal organization of the SR concerning HIV vulnerability provide a coherent overview of the potential central and peripheral elements, enabling an understanding of how the group perceives and positions itself regarding these issues.

### Study limitations

One limitation of the study is that qualitative research does not allow for generalizations. Identifying the living conditions of individuals within a historical, social, cultural, and healthcare context marked by prejudice and stigma is challenging, as social thought is anchored in cis-heteronormative norms, leading to judgments that contribute to the marginalization and exclusion of transgender women.

### Contributions to the Field of Nursing, Health, or Public Policy

The study facilitated the identification of the structures of SR, with scientifically relevant implications for the field of nursing. SR do not establish definitive concepts or predeterminations but instead allow for contrasting social and ideological intentions in the relationships between transgender women and society in addressing the epidemic. The recognition of representational content can stimulate discussions among healthcare teams about the quality of care provided, help identify which policies and local partnerships are best suited to respond to the needs of transgender women receiving care, and, consequently, improve health services, particularly for populations in vulnerable situations.

## FINAL CONSIDERATIONS

The TSR enables the elucidation of the systems of meaning produced and shared by transgender women within a specific social context. Based on the results obtained, it was possible to observe representational content regarding HIV vulnerability among transgender women related to prevention, prostitution as a profession, prejudice, and risky behavior, evidenced in the central nucleus of the representation due to the denial of social rights, such as access to healthcare, education, and employment, within a context of marginalization, transphobia, and social exclusion. These contents are supported by the peripheral elements “social vulnerability” and “internal suffering”, which emphasize the individual and social aspects of HIV exposure.

## Data Availability

Not applicable.
