# Supplementary material for: Representation and social vulnerability to HIV in transgender people
Source: Rev Bras Enferm. 2025 Aug 8;78(Suppl 2):e20230353. doi: 10.1590/0034-7167-2023-0353 (PMC12334129; doi:10.1590/0034-7167-2023-0353)
Supplement: Translation

# Translation: Representação e vulnerabilidade social ao HIV em pessoas transgênero

**DOI:** 10.1590/0034-7167-2023-0353pt

**Authors:** Karla Romana Ferreira de Souza, Elizandra Cassia da Silva Oliveira, Carla Andreia Alves de Andrade, Sérgio Corrêa Marques, Fátima Maria Leite Cruz, Aurélio Molina da Costa, Fátima Maria Abrão da Silva

**Affiliations:** I Universidade de Pernambuco. Recife, Pernambuco, Brasil; II Universidade Estadual do Rio de Janeiro. Rio de Janeiro, Rio de Janeiro, Brasil; III Universidade Federal de Pernambuco. Recife, Pernambuco, Brasil; IV Universidade de Leeds. Leeds, Reino Unido

**Keywords:** Enfermagem, Pessoas Transgênero, Vulnerabilidade Social, HIV, Teoria Social

## Abstract

**Objetivos:**

analisar as representações sociais da vulnerabilidade ao HIV em mulheres transexuais.

**Métodos:**

estudo qualitativo, fundamentado na Teoria das Representações Sociais, utilizando a abordagem estrutural. A pesquisa foi realizada em um hospital de referência em cirurgias de redesignação, com 100 mulheres autodeclaradas transexuais. Utilizou-se a técnica de Evocações Livres de Palavras ao termo indutor “exposição ao HIV em mulheres transexuais” para obtenção dos conteúdos da representação. A análise dos dados foi realizada por meio da técnica do quadro de quatro casas, instrumentalizada pelo *software Interface de R pour les Analyses Multidimensionnelles de Textes et de Questionnaires*, versão 0.7 alpha 2.

**Resultados:**

Identificou-se, no possível núcleo central, os termos “prevenção, prostituição como profissão, preconceito e comportamento de risco”. Questões sociais e individuais foram destacadas como elementos importantes no processo de vulnerabilidade.

**Conclusões:**

As representações sociais da vulnerabilidade ao HIV em mulheres transexuais estão pautadas em fatores estruturais causadores de desigualdade, exclusão, preconceito e discriminação.

## INTRODUÇÃO

As situações de vulnerabilidade ao vírus da imunodeficiência humana (HIV) são um dos grandes desafios para a sua disseminação epidêmica, por serem consideradas responsáveis pelo processo de propagação da epidemia, impactando de forma diferente as diversas populações. Dados do *Joint United Nations Program on HIV/AIDS (UNAIDS)*, de 2019, mostram que, globalmente, a população transexual tem 12 vezes mais chance de infecção pelo HIV do que a população em geral^(1)^.

Os estudos sobre vulnerabilidade buscam responder à percepção da chance de exposição das pessoas ao HIV e ao adoecimento pela Síndrome da Imunodeficiência Adquirida (AIDS), considerando que não se trata apenas de um conjunto de aspectos individuais, mas também coletivos e contextuais, que acarretam maior suscetibilidade à infecção e ao adoecimento. Além disso, há uma maior ou menor disponibilidade de recursos em todas as esferas federais, estaduais e municipais, que garantam proteção nessas diferentes esferas governamentais. A análise de vulnerabilidade busca integrar três dimensões: individual, social e programática ou institucional, interdependentes, possibilitando compreender os aspectos da vida das pessoas, das comunidades e das nações que as tornam mais ou menos suscetíveis à infecção pelo HIV, ao adoecimento ou à morte por AIDS^(2,3)^.

Ressaltamos que as mulheres transexuais frequentemente são submetidas a processos de vulnerabilização, intensificados pela sobreposição de preconceitos derivados de sua identidade de gênero. Essa dinâmica, caracterizada como transfobia, não apenas impacta profundamente a vida e a saúde dessas mulheres, mas também as coloca em um estado de constante precariedade social no processo saúde-doença. A transfobia não é apenas um fenômeno isolado de discriminação, mas um sistema estrutural que reforça marginalizações múltiplas e desigualdades, aumentando significativamente a vulnerabilidade ao HIV^(4)^.

Pesquisas envolvendo as questões da vulnerabilidade ao HIV têm o propósito de analisar a disseminação da epidemia e seu impacto nas diversas populações. Seus resultados possibilitam identificar e reconhecer as diferenças e especificidades desse processo, essenciais para o planejamento e a implementação de políticas e programas voltados ao atendimento dos grupos mais vulneráveis à exposição ao HIV^(5)^.

Atualmente, são incipientes os estudos que abordem a discussão da disseminação do HIV a partir das condições de vulnerabilidade da doença e dos significados construídos para enfrentar essa realidade. Buscam-se compreender o universo representacional e consensual das mulheres transexuais em relação ao HIV. Considerando que o HIV/Aids é um fenômeno social que vem sofrendo transformações ao longo do tempo, dar voz às pessoas que recebem os cuidados de saúde é fundamental para conhecer suas necessidades, a partir das experiências construídas no cotidiano, através das atitudes, comportamentos e sentimentos, contribuindo para ações de promoção e prevenção mais adequadas e satisfatórias^(6,7)^.

Compreender a epidemia de HIV/Aids, que surgiu em 1990, como uma questão individual dependerá do contexto socioeconômico e cultural no qual o indivíduo está inserido. As discussões, anteriormente debatidas sob a ótica do comportamento individual, classificavam “populações de risco”, o que ainda gera vulnerabilidade social, estigmas e preconceitos. Hoje, essas discussões deslocam-se para questões de vulnerabilidade social, permitindo debates sobre direitos humanos e saúde^(8)^.

A Teoria das Representações Sociais (TRS), com base nos pressupostos de Moscovici, possui três abordagens teórico-metodológicas diferentes para estudar o fenômeno das Representações Sociais (RS): a abordagem processual (ou culturalista), desenvolvida por Denise Jodelet; a abordagem estrutural, proposta por Jean-Claude Abric; e a abordagem societal, defendida por Willem Doise^(9)^.

Refletir sobre as situações de vulnerabilidade das mulheres transexuais ao HIV pode trazer implicações importantes, especialmente no que diz respeito à prevenção nessa população. A abordagem estrutural da TRS, que corresponde à teoria do núcleo central, desenvolvida por Jean-Claude Abric, permite vincular as relações que as mulheres transexuais estabelecem com o cotidiano de suas práticas e as significações que atribuem às condições de vulnerabilidade ao HIV.

## OBJETIVOS

Analisar as RS da vulnerabilidade ao HIV em mulheres transexuais.

## MÉTODOS

### Aspectos Éticos

O projeto deste estudo foi aprovado pelo Comitê de Ética em Pesquisa e desenvolvido de acordo com as diretrizes da Resolução nº 466/2012 do Conselho Nacional de Saúde. Todos os participantes assinaram o Termo de Consentimento Livre e Esclarecido.

### Tipo de Estudo

Trata-se de um estudo qualitativo, conduzido em conformidade com as recomendações do *Consolidated Criteria for Reporting Qualitative Research (COREQ)*.

### Referencial Teórico-Metodológico

Este estudo tem como referencial teórico a TRS de Serge Moscovici, com ênfase na Teoria do Núcleo Central (TNC). Esta teoria compreende as RS construídas por dois processos principais: ancoragem e objetivação. A ancoragem consiste na incorporação ou assimilação de novos elementos de um objeto em um sistema de categorias já familiar ao sujeito, disponível na memória. Já a objetivação busca transformar o que era abstrato em concreto, relacionando com o mundo físico o que já estava na mente, unindo a ideia de não familiaridade com a de realidade^(9)^.

A abordagem teórico-metodológica utilizada para estudar o fenômeno das RS foi a estrutural, proposta por Jean-Claude Abric, que examina os conteúdos das RS e a forma como eles se organizam, considerando questões de estabilidade e mudança, bem como sua relação com a prática social do grupo em questão. Existe um núcleo central em torno do qual a RS se estrutura, determinado pela natureza do objeto em estudo, pela forma como o grupo se relaciona com ele e pelo sistema de valores e normas sociais que compõem o meio ideológico. O núcleo central é o elemento mais estável da representação e, portanto, resistente às mudanças. Existem também elementos periféricos que compõem a RS, organizados em torno do núcleo central. Esses elementos são mais acessíveis, dinâmicos e concretos; integram as experiências cotidianas e são mais individualizados e localizados^(10)^.

### Cenário do Estudo

A pesquisa foi conduzida no Espaço de Cuidado e Acolhimento para Pessoas Transexuais, localizado em um hospital universitário de referência na região Norte-Nordeste do Brasil em cirurgias de redesignação. O convite para participação na pesquisa foi feito na sala de espera do hospital, enquanto as mulheres transexuais aguardavam atendimento. Uma vez aceito o convite, as participantes foram conduzidas para uma sala reservada pela instituição, onde foram realizadas entrevistas individuais.

### Fonte de Dados

O estudo contou com a participação de 100 mulheres autodeclaradas transexuais. As participantes foram selecionadas por amostragem de conveniência, a partir dos seguintes critérios de inclusão: ser transexual e se identificar como sendo de gênero feminino. A aproximação com as participantes foi facilitada pela equipe multiprofissional. Foram excluídas pessoas com deficiência auditiva, visto que a pesquisadora não possuía domínio da Língua Brasileira de Sinais.

## RESULTADOS

A caracterização sociodemográfica e clínica dos participantes revelou que a faixa etária predominante é de 18 a 29 anos, correspondendo a 54 (54%) participantes. Em relação à escolaridade, 65 (65%) relataram possuir ensino médio. Quanto à renda, 44 (44%) afirmaram não ter renda, e, no que diz respeito ao estado civil, 70 (70%) disseram estar solteiras.

O produto das evocações livres traduz a estrutura das RS da vulnerabilidade ao HIV em mulheres transexuais. Foram definidos os seguintes parâmetros para que o *software* processasse os dados, sendo necessário, previamente, estabelecer: a frequência mínima de palavras, definida em 11, com exclusão dos termos evocados abaixo dessa frequência; cálculo da frequência média de evocação, que correspondeu a 28,8. Com base nessas frequências, o programa calculou a média das Ordens Médias de Evocação (OME), que foi de 2,88, ajustada para 2,9.

Ao evocarmos o objeto social “exposição ao HIV em mulheres transexuais”, com o objetivo de compreendê-lo, outros objetos se misturaram ao objeto central da pesquisa. Isso significa que as participantes não se concentraram exclusivamente na exposição ao HIV em mulheres transexuais, conforme questionado, mas se relacionaram aos discursos a partir do referencial de suas histórias de vida, remetendo a outros temas que estão inseridos no processo de exposição ao HIV em mulheres transexuais. Isso demonstra que nenhum objeto representacional é isolado de outros objetos, articulando-se com eles, conforme descrito no Quadro 1.

**Quadro 1:** Quadro de Quatro Casas sobre o termo indutor “Exposição ao HIV em mulheres transexuais”, com base no relatório fornecido pelo programa

Frequência mínima: 11						
**Frequência intermediária: 28,8**						
**Ordem média: 11**						
**Ordem média < 11**				**Ordem média > = 11**		
**1º quadrante - Núcleo central**				**2º quadrante - Primeira periferia**		
**F > = 28,8**	Prevenção	60	2,8	Vulnerabilidade social	53	3,3
	Prostituição como profissão	46	2,7	Sofrimento intemo	52	3,1
	Preconceito	42	2,7			
	Comportamento de risco	34	2,9			
**3º quadrante - Zona de contraste**				**4º quadrante - Segunda periferia**		
**F < = 28,8**	Tentar ser positivo	27	2,7	Drogas	16	3,2
	Sexo sem camisinha	23	2,8	Aidético	16	3,4
	Falta de oportunidade de trabalho	13	2,8	Confiança	15	3,1
	Fragilidade das políticas públicas	12	2,7	Desinformação	12	3,8
	Sem apoio familiar	11	2,7			

*Fonte: Dados de pesquisa processados no software IRaMuTeQ®.*

O resultado do relatório de frequência de evocações (F), em resposta à expressão indutora “exposição ao HIV em mulheres transexuais”, apresenta, no quadrante superior esquerdo, os prováveis elementos centrais, que são as palavras mais mencionadas pelas mulheres transexuais e definidas como as mais importantes: prevenção, prostituição como profissão, preconceito e comportamento de risco. O termo mais frequente, ou seja, o mais evocado pelas participantes, foi “prevenção”, mencionado 60 vezes (com frequência de 2,8), seguido de “prostituição como profissão”, mencionado 46 vezes (frequência de 2,7), “preconceito”, mencionado 42 vezes (frequência de 2,7) e “comportamento de risco”, mencionado 34 vezes (frequência de 2,9). A presença desses termos no provável núcleo central da RS sobre a exposição ao HIV em mulheres transexuais reflete a importância dada pelas participantes à prevenção ao HIV. No entanto, reconhecem-se situações de vulnerabilidade social, considerando que muitas dessas mulheres são levadas à prostituição como profissão devido ao preconceito. A presença da expressão “comportamento de risco” demonstra o reconhecimento das situações de exposição ao HIV na perspectiva da vulnerabilidade individual.

Diante do exposto, observa-se que todos os termos do possível núcleo central estão relacionados e sugerem situações de vulnerabilidade às quais as mulheres transexuais estão sujeitas em seu cotidiano. Destacamos que a ausência de uma afirmação segura sobre o núcleo central deveu-se à não aplicabilidade do teste de centralidade para confirmação do núcleo central, devido à limitação de tempo para a conclusão da pesquisa.

Compondo o sistema periférico (primeira e segunda periferias), encontram-se os seguintes elementos: na primeira periferia, localizada no quadrante superior direito, “vulnerabilidade social” foi evocado 53 vezes (com frequência de 3,3) e “sofrimento interno” foi mencionado 52 vezes (com frequência de 3,1). Ao avaliar a primeira periferia, o termo “vulnerabilidade social” destaca-se pela alta frequência (60), reforçando os conteúdos presentes no núcleo central, expressos através dos termos “vulnerabilidade social” e “sofrimento interno”, que remetem à dimensão afetivo-atitudinal relacionada à vulnerabilidade social. Os termos expressos na segunda periferia apresentam alguns atributos negativos, como “drogas”, “aidético”, “confiança” e “desinformação”, remetendo a uma dimensão mais conceitual, além de afetiva e atitudinal, respectivamente, decorrente da construção histórica acerca do HIV/Aids e das mulheres transexuais, relacionando suas vulnerabilidades individuais.

Na segunda periferia, no quadrante inferior direito, os elementos de maior destaque são: drogas, aidético, confiança, desinformação, objetos cortantes, contaminação com agulha, falta de afeto, desconfiança e beijo. Os elementos de maior relevância são “drogas” e “aidético”, devido às maiores frequências e menores OME neste quadrante. O termo “drogas” está fortemente vinculado à AIDS no início da epidemia no Brasil, enquanto a palavra “aidético”, muito utilizada no início da epidemia, originou estigma e preconceito. Esses termos remetem à construção histórica e cultural da doença. Pode-se, então, afirmar que esses elementos indicam os diversos sentidos atribuídos pelas mulheres transexuais à vulnerabilidade ao HIV.

Os elementos presentes na zona de contraste, no quadrante inferior esquerdo, são termos com baixa frequência e baixa OME, ou seja, aqueles menos mencionados, mas prontamente evocados pelas participantes, sendo, portanto, considerados importantes para as poucas pessoas que os evocaram. São eles: “tentar ser positivo”, “sexo sem camisinha”, “falta de oportunidade de trabalho”, “fragilidade nas políticas públicas” e “sem apoio familiar”.

O termo “tentar ser positivo” foi o mais expressivo da zona de contraste, devido à maior frequência entre os elementos deste quadrante, evidenciando a importância dos mecanismos de enfrentamento das situações de vulnerabilidade. Os demais termos reforçam e justificam os elementos evocados no provável núcleo central.

## DISCUSSÃO

No possível núcleo central da RS sobre a exposição ao HIV em mulheres transexuais encontram-se conteúdos representacionais que apresentam um atributo positivo relacionado à atitude de prevenção ao HIV/Aids e três atributos negativos. O atributo positivo refere-se à atitude de prevenção da doença, associada e condicionada ao uso do preservativo durante as práticas sexuais; já os aspectos negativos estão vinculados a questões socioculturais, econômicas e psicossociais, expressos pelas palavras “prostituição como profissão”, “preconceito” e “comportamento de risco”.

O núcleo central é formado por elementos normativos e funcionais. Assim, considera-se que o conteúdo “prevenção” seja a dimensão funcional, por estar associado às condutas relativas às práticas sociais. Por outro lado, os elementos “prostituição como profissão”, “preconceito” e “comportamento de risco” são normativos, uma vez que estão relacionados à história e à ideologia do grupo social, ligadas aos sistemas de valores sociais. Eles privilegiam os julgamentos, estereótipos e opiniões admitidos pelo sujeito ou grupo social no qual ele se insere^(11)^.

Dentre os elementos preventivos, a utilização de preservativos durante as relações sexuais, em um estudo realizado no Brasil com profissionais do sexo, também foi citada como a forma mais conhecida e utilizada para se proteger contra o HIV/Aids^(12)^. Todavia, a prevenção combinada tem sido uma nova abordagem estabelecida pelo Ministério da Saúde para prevenção ao HIV, incluindo a profilaxia pré-exposição (PrEP) e a profilaxia pós-exposição (PEP), além da distribuição de preservativos masculinos e/ou femininos, géis lubrificantes, testagem regular do anti-HIV sob livre demanda e aconselhamento^(13)^.

Elementos de contraste, como “tentar ser positivo”, “sexo sem camisinha”, “falta de oportunidade de trabalho”, “fragilidade nas políticas públicas” e “sem apoio familiar” foram identificados nos resultados da pesquisa. Apesar de menos frequentes, são relevantes para alguns participantes e estão relacionados aos temas centrais de prevenção, prostituição como profissão, preconceito e comportamento de risco. Mulheres transexuais reconhecem a necessidade de superar os desafios de sobreviver em um contexto social marcado pela transfobia, que afeta profundamente seu direito à cidadania. Promover iniciativas que restaurem a autoestima, reconstruam redes de apoio e afirmem projetos de vida seguros é crucial para que tentem ser positivas. Este contexto de vulnerabilidade social, exacerbado pela transfobia, preconceito, falta de apoio familiar e oportunidades de trabalho, muitas vezes leva à prostituição como fonte de renda, resultando em negociações arriscadas sobre o uso de preservativos, levando ao sexo sem camisinha e gerando comportamentos de risco. É evidente a fragilidade das políticas públicas na promoção de medidas eficazes de prevenção específicas para mulheres transexuais^(14)^.

Portanto, a discussão de políticas públicas para o aumento da disponibilidade de estratégias de prevenção é importante e necessária, principalmente na qualificação profissional. Um estudo realizado no Brasil revelou um déficit de conhecimento dos estudantes de medicina e enfermagem quanto ao uso da PrEP e PEP, assim como em estudos realizados no Nepal, Gana e Etiópia. Torna-se necessária uma sinergia para a melhoria da qualidade dos serviços de saúde, especialmente na qualificação profissional^(15)^.

A utilização de hormônios no processo de transformação corporal pode provocar disfunção erétil, aumentando a probabilidade de a mulher transexual desempenhar o papel receptivo nas relações sexuais. Há evidências das dificuldades na negociação do uso de preservativos, uma vez que cabe ao homem decidir ou não por seu uso. O sexo anal desprotegido representa um elevado risco de transmissão do HIV. As mulheres transexuais enfrentam dificuldades em exigir o uso de preservativos nas relações sexuais com homens^(16)^.

Quanto às questões socioculturais, econômicas e psicossociais enfrentadas por essa população, muitas delas são fruto histórico de uma suposta verdade codificada para formar corpos heterossexuais-normativos, que resultam em violências, invisibilidade e vulnerabilidade para aqueles que apresentam discordância em relação ao padrão social estabelecido. Essas pessoas são vistas como anomalias, desvios ou doenças devido ao modo como se comportam, o que desperta muitas interrogações, estranhamentos e incômodos^(17)^.

Estudos revelam que o estigma e a discriminação desempenham um papel importante, influenciando comportamentos, práticas e atitudes em relação ao HIV. Esses fatores podem limitar o acesso a recursos socioeconômicos, educacionais e laborais, assim como o acesso a serviços de prevenção, tornando-se dois dos principais fatores associados às altas prevalências de infecção pelo HIV^(18)^. Um exemplo disso é o estudo realizado na Argentina, que revelou que 40,7% das mulheres transgênero evitam o uso de serviços de saúde devido à sua identidade de gênero, por experiências de discriminação por parte de profissionais de saúde ou pacientes, além de agressões por policiais^(19)^.

No sistema periférico da RS analisada, a vulnerabilidade social e o sofrimento interno reforçam a análise das relações entre intersubjetividades, contextos sociais, diálogos e conflitos que permeiam o cotidiano das mulheres trans em relação aos elementos centrais. Conforme Abric^(10)^, esses são os componentes mais acessíveis de uma representação, os mais suscetíveis a mudanças. Eles atualizam e contextualizam as determinações normativas e consensuais, promovendo a interface entre a realidade concreta e o sistema central.

A população transexual enfrenta diversas situações de vulnerabilidade social, evidenciadas pela privação do convívio familiar e escolar, geralmente marcada pelo não acolhimento e pela violência, o que leva a sofrimentos internos diversos. Segundo a Rede Nacional de Pessoas Trans e a Associação Nacional de Transexuais e Travestis do Brasil (ANTRA)^(20)^, 82% das mulheres transexuais abandonaram o ensino médio entre os 14 e 18 anos, e 90% acabam na prostituição.

As pessoas transexuais são frequentemente excluídas de vários grupos sociais, seja pelos próprios pais, famílias ou pela sociedade, por romperem com o sistema da cis-heteronormatividade. A quebra desses padrões sociais de gênero gera preconceito e discriminação, contribuindo para uma série de efeitos negativos na saúde mental. Dados sugerem que o índice de suicídio entre pessoas cisgêneras apresenta um percentual de 4,6%, enquanto o risco entre indivíduos transgêneros sobe para 41%. Ressalta-se que jovens de minorias de gênero são um grupo particularmente vulnerável ao suicídio^(21)^.

Um estudo realizado no Brasil constatou que, dentre 154 participantes transexuais, 48,3% apresentavam ideação suicida e 23,8% haviam tentado suicídio, com níveis mais altos de ideação e tentativas observados em pessoas com disforia de gênero do que na população geral. Da mesma forma, estudos realizados na Suíça revelaram que a prevalência de ideação suicida foi quase sete vezes e meia maior entre os entrevistados transexuais^(22)^.

Os conteúdos e a organização interna da RS sobre a vulnerabilidade ao HIV fornecem um panorama coerente entre os possíveis elementos centrais e periféricos, permitindo entender como o grupo pensa e se posiciona a respeito de si mesmo.

### Limitações do estudo

Como limitação do estudo, cabe ressaltar que um trabalho de natureza qualitativa não permite fazer generalizações. Identificar as condições de vida de pessoas inseridas em um contexto histórico, social, cultural e de atenção à saúde permeado por preconceitos e estigmas é um desafio, pois o pensamento social está ancorado às normas cis-heteronormativas, gerando julgamentos que contribuem para o processo de marginalização e exclusão das mulheres transexuais.

### Contribuições para a Área da Enfermagem, Saúde ou Políticas Públicas

O estudo respaldou a identificação das estruturas das RS, com implicações científicas relevantes para a ciência da enfermagem. As RS não estabelecem conceitos ou predeterminações definitivas, mas possibilitam contrastar intenções sociais e ideológicas nas relações entre as mulheres transexuais e a sociedade para o enfrentamento da epidemia. Considera-se que o reconhecimento dos conteúdos representacionais pode suscitar discussões nas equipes de saúde sobre a qualidade do acompanhamento oferecido, identificar quais políticas e parcerias locais são mais adequadas para responder às demandas das mulheres transexuais assistidas e, assim, aperfeiçoar os cuidados de saúde, especialmente em populações em situações de vulnerabilidade.

## CONSIDERAÇÕES FINAIS

A TRS possibilita esclarecer os sistemas de significação que são produzidos e partilhados pelas mulheres transexuais, inseridas em um contexto social específico. Diante dos resultados encontrados, foi possível observar conteúdos representacionais da vulnerabilidade ao HIV em mulheres transexuais relacionados à prevenção, prostituição como profissão, preconceito e comportamento de risco, evidenciados no núcleo central da representação, em decorrência da negação de direitos sociais, como acesso à saúde, educação e emprego, devido ao contexto de marginalização, transfobia e exclusão social. Esses conteúdos são corroborados pelos elementos periféricos “vulnerabilidade social” e “sofrimento interno”, que enfatizam os aspectos individuais e sociais da exposição ao HIV.

## Data Availability

Não se aplica.
